# Mutations in *FKBP10* Cause Recessive Osteogenesis Imperfecta and Bruck Syndrome

**DOI:** 10.1002/jbmr.250

**Published:** 2010-09-13

**Authors:** Brian P Kelley, Fransiska Malfait, Luisa Bonafe, Dustin Baldridge, Erica Homan, Sofie Symoens, Andy Willaert, Nursel Elcioglu, Lionel Van Maldergem, Christine Verellen-Dumoulin, Yves Gillerot, Dobrawa Napierala, Deborah Krakow, Peter Beighton, Andrea Superti-Furga, Anne De Paepe, Brendan Lee

**Affiliations:** 1 Department of Molecular and Human Genetics, Baylor College of MedicineHouston, TX, USA; 2 Howard Hughes Medical InstituteHouston, TX, USA; 3 Center for Medical Genetics, Ghent University HospitalGhent, Belgium; 4 Division of Molecular Pediatrics, Centre Hospitalier Universitaire VaudoisLausanne, Switzerland; 5 Department of Pediatric Genetics, Marmara University Medical FacultyIstanbul, Turkey; 6 Centre de Génétique Humaine, Universite de LiègeLiège, Belgium; 7 Center for Human Genetics, Cliniques Universitaires St Luc and University of Louvain Medical SchoolBrussels, Belgium; 8 Medical Genetics Institute, Cedars-Sinai Medical Center, David Geffen School of Medicine at UCLALos Angeles, CA, USA; 9 Division of Human Genetics, University of CapetownObservatory, South Africa

**Keywords:** OSTEOGENESIS IMPERFECTA, BRUCK SYNDROME, *FKBP10* (ALSO KNOWN AS *FKBP65*), BRITTLE BONE DISEASE, COLLAGEN

## Abstract

Osteogenesis imperfecta (OI) is a genetic disorder of connective tissue characterized by bone fragility and alteration in synthesis and posttranslational modification of type I collagen. Autosomal dominant OI is caused by mutations in the genes (*COL1A1* or *COL1A2*) encoding the chains of type I collagen. Bruck syndrome is a recessive disorder featuring congenital contractures in addition to bone fragility; Bruck syndrome type 2 is caused by mutations in *PLOD2* encoding collagen lysyl hydroxylase, whereas Bruck syndrome type 1 has been mapped to chromosome 17, with evidence suggesting region 17p12, but the gene has remained elusive so far. Recently, the molecular spectrum of OI has been expanded with the description of the basis of a unique posttranslational modification of type I procollagen, that is, 3-prolyl-hydroxylation. Three proteins, cartilage-associated protein (CRTAP), prolyl-3-hydroxylase-1 (P3H1, encoded by the *LEPRE1* gene), and the prolyl *cis-trans* isomerase cyclophilin-B (PPIB), form a complex that is required for fibrillar collagen 3-prolyl-hydroxylation, and mutations in each gene have been shown to cause recessive forms of OI. Since then, an additional putative collagen chaperone complex, composed of FKBP10 (also known as FKBP65) and SERPINH1 (also known as HSP47), also has been shown to be mutated in recessive OI. Here we describe five families with OI-like bone fragility in association with congenital contractures who all had *FKBP10* mutations. Therefore, we conclude that *FKBP10* mutations are a cause of recessive osteogenesis imperfecta and Bruck syndrome, possibly Bruck syndrome Type 1 since the location on chromosome 17 has not been definitely localized. © 2011 American Society for Bone and Mineral Research.

## Introduction

**Fig. 1:**
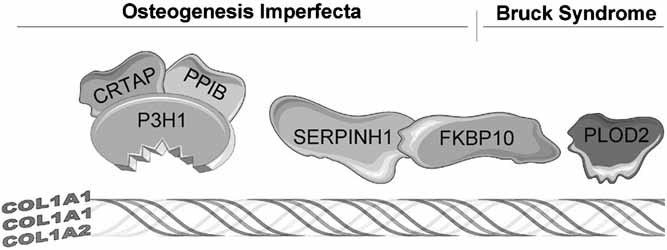
Components of the posttranslational modification machinery of type I collagen causing osteogenesis imperfecta and Bruck syndrome. Figure depicts proteins involved in posttranslational modification of type I collagen and mutated in osteogenesis imperfecta (OI) or Bruck syndrome. Mutations in genes (*COL1A1* and *COL1A2*) encoding the α_1_ and α_2_ chains of type I procollagen most commonly result in dominant OI and rarely recessive OI. Other rare causes of recessive OI have been shown to be caused by mutations in genes encoding the CRTAP/P3H1/cyclophilin B or SERPINH1/FKBP10 protein complexes. These proteins modify collagen by 3-prolyl-hydroxylation and serve as endoplasmic reticulum chaperones, respectively. Bruck syndrome is also caused by mutations in genes coding for proteins involved in posttranslational modification of collagen, specifically lysyl hydroxylation. The figure was produced using Servier Medical Art: http://www.servier.com/Smart/ImageBank.aspx?id_=729.

Type I procollagen undergoes multiple intracellular and extracellular posttranslational modifications. Mutations in genes encoding type I procollagen or proteins responsible for posttranslational modifications of the type I collagen heterotrimer may result in the brittle bone disorder osteogenesis imperfecta (OI). Classic OI, an autosomal dominant heritable disorder, has been linked to mutations in the *COL1A1* and *COL1A2* genes, which code for procollagen α components of the type I collagen heterotrimer [α_1_(I)_2_ and α_2_(I)_1_]. After assembly of type I procollagen in the endoplasmic reticulum (ER), two α_1_ chains [α_1_(I)_2_] and one α_2_ chain [α_2_(I)_1_] become noncovalently linked at their carboxyl-terminus. This is followed shortly by interchain disulfide bond formation by protein-disulfide isomerase (PDI). The unfolded chains then undergo 4-prolyl-hydroxylation, 3-prolyl-hydroxylation, and lysyl hydroxylation that is then followed by assembly of the triple helix. Recently, new proteins involved in posttranslational modification of collagen have been implicated in recessively inherited forms of OI (Fig. 1). Specifically, a complex responsible for 3-prolyl-hydroxylation composed of cartilage-associated protein (CRTAP)/prolyl-3-hydroxylase (P3H1, encoded by the *LEPRE1* gene)/cyclophilin B (PPIB) has been implicated in causing recessive OI.(1–4) Furthermore, mutations in all three proteins now have been shown to cause recessive forms of OI in part by leading to loss of hydroxylation at proline residue 986 of the α_1_(I) triple-helical domain and subsequent collagen overmodification. Finally, proteins responsible for chaperoning collagen through the ER have been implicated in recessive OI cases that do not show evidence of collagen overmodification. The FK506 binding protein 10 (FKBP10) and SERPINH1 ER-resident chaperone complex have specifically been found to cause recessive OI.(5,6)

Bruck syndrome is a rare autosomal recessive disorder that is phenotypically related to OI. The characteristics of Bruck syndrome include bone fragility, congenital joint contractures with webbing (pterygia), scoliosis, and osteoporosis (Table 1). Bruck syndrome is further delineated into type 1 and type 2, which are phenotypically indistinguishable, and discussed in more detail below. Previously, it has been suggested that this disorder is related to decreased posttranslational cross-linking of collagen fibrils.(7). Collagen typically is stabilized into fibrils by lysyl oxidation of lysine to aldehyde allysine and hydroxylysine to aldehyde hydroxyallysine.(8–11) By either pathway, mature cross-links of hydroxylysylpyridinoline or lysylpyridinoline are formed.(12) Furthermore, the molecular defect in Bruck syndrome was described to be associated with aberrant cross-linking of bone collagen owing to underhydroxylation, whereas cartilage and ligamentous collagen maintained normal cross-linking.(7) A single consanguineous family was shown to have linkage to 17p12, which was the first proposed site for the gene encoding for the bone-specific telopeptide lysyl hydroxylase.(7,13) However, in 2003, Van der Slot and colleagues described two independent families with mutations in the *PLOD2* gene who had been diagnosed with Bruck syndrome by biochemical analysis of their bone showing decreased pyridinoline cross-linking.(14) PLOD2 is a member of the PLOD family of proteins responsible for lysyl hydroxylation and in which mutations have been shown to cause Ehlers-Danlos syndrome as well as Bruck syndrome type 2. PLOD2 specifically functions as a telopeptide lysyl hydroxylase. However, these cases showed no linkage to the previously described chromosome 17p12 site because the *PLOD2* gene maps to 3q23-24. Van der Slot and colleagues thus proposed that cases of Bruck syndrome linked to 17p12 and those linked to 3q23-24 (caused by mutations in the *PLOD2* gene) be termed *Bruck syndrome 1* and *Bruck syndrome 2*, respectively.(14)

**Table 1:** Clinical and Phenotypic Comparison of Bruck Syndrome and Osteogenesis Imperfecta

	Classic osteogenesis imperfecta type 1	Osteogenesis imperfecta type 3	Bruck syndrome^a^
Bone fragility	+	++	++
Osteoporosis	+	++	++
Blue/gray sclera	+/−	+/−	+/−
Dentinogenesis imperfecta	+/−	+/−	−
Hearing loss	++	+/−	−
Congenital ptygeria	−	−	+++

a Type 1 and type 2 Bruck syndrome are clinically indistinguishable.

Previous studies have suggested that FKBP10 may have multiple innate functions, including peptidylprolyl *cis-trans* isomerase (PPIase) and chaperone capability. Further, these studies have elucidated that the PPIase activity of FKBP10 functions in folding of proline-rich tropoelastin.(15) With regard to the folding of the type I collagen heterotrimer, the activity of the FKBP10 PPIase may be marginal.(16) In vitro, FKBP10 in conjunction with SERPINH1 has been shown to act as chaperone of the collagen heterotrimer.(17) Further, *SERPINH1* null fibroblasts have demonstrated deficient fibrillogenesis with aggregation of type I collagen in the ER.(18) Loss of the FKBP10/SERPINH1 complex owing to deletion of the *SERPINH1* gene was shown originally to cause recessive OI in a dachshund model.(19) However, *SERPINH1* mutations in humans had not been identified until the very recent report of a single child with the phenotype of OI type 3. The child was found to have a homozygous missense mutation in the coding region of *SERPINH1.*(5)

Novel mutations in *FKBP10* have been reported recently to cause a recessive OI-like phenotype in humans. In a cohort of five consanguineous Turkish families and one Mexican-American family, *FKBP10* mutations were found to cause moderately severe recessive OI. The OI phenotype demonstrated recurrent long bone fractures beginning in infancy, with eventual need for a wheelchair in early childhood. Affected patients further demonstrated progressive kyphoscoliosis, severe osteopenia, ligamentous laxity, grayish-white sclera, normal hearing, and normal teeth. An iliac crest biopsy demonstrated a “fish scale” pattern in one patient under polarized-light histology.(6)

Taking into account the results of Alanay and colleagues and Christensen and colleagues, there are now three classes of mechanisms for generating an OI phenotype.(5,6) First, mutations in the *COL1A1* or *COL1A2* gene may cause quantitative and/or qualitative defects in type I procollagen leading to dominantly inherited OI.(22,23) Second, posttranslational modification of type I procollagen is also critical to forming properly functioning collagen helices. Disease-causing mutations have been found in all three genes coding for the P3H1/CRTAP/cyclophilin B protein complex, which is responsible for fibrillar collagen prolyl-3-hydroxylation. Finally, with the discovery of disease-causing *FKBP10* and *SERPINH1* mutations, defects in ER chaperone protein complexes have been noted to cause recessive OI.

Here we present five families with a moderately severe OI phenotype in association with some patients having congenital contractures of the knees, ankles, and/or elbows. In all but one family, at least one child eventually was diagnosed with Bruck syndrome type 1. In one family we found significant evidence of variable expressivity, with one child with Bruck syndrome, whereas his sister presented with conventional OI type 3. Diagnosis of Bruck syndrome depended on clinical and laboratory findings, including urine lysyl oxidation assays, negative screens for mutations in the *PLOD2* gene, and the finding of congenital contractures in the presence of bone fragility. Therefore, we propose that mutations in *FKBP10* may be responsible for cases of type 1 Bruck syndrome presenting with a recessive, moderately severe OI phenotype. Further, we propose that given phenotypic variations within family members having the same genotype, Bruck syndrome type 1 in fact may be a variant of the larger OI spectrum.

## Methods

### Patients

A total of 67 patients were selected for screening based on reported consanguinity, familial origination in a region known for increased rates of consanguinity, multiple affected siblings from the same family, failure to identify mutations in the *COL1A1* or *COL1A2* gene, and absence of disease-causing mutations in known genes related to recessive OI, including *CRTAP* and *LEPRE1*. Of the 67 patients screened, 57 patients were index cases, and 10 were family members related to the index cases. With institutional review board approvals, blood, fibroblasts, or tissue was collected from affected individuals, and DNA was prepared by standard protocols. Patient histories were reviewed following direct history/examination by the clinical coauthors. The age of presentation listed in each case study is the most recent patient age for clinical data review.

### PCR and sequence analysis

The 10 exons of *FKBP10*, as well as surrounding intronic regions, were amplified from genomic DNA patient samples by PCR and analyzed by fluorescent dye terminator sequencing (Agencourt Bioscience Services, a Division of Beckman Coulter Genomics, Danvers, MA, USA) or by sequencing on the ABI3730XL apparatus from Applied BioSystems, Inc. (Foster City, CA, USA). Results were analyzed using Sequencher 4.8 software (Gene Codes Corporation, Ann Arbor, MI, USA) or SeqScape software (Applied BioSystems). Patient sequences were compared with the Ensembl gene sequence ENSG00000141756 for *FKBP10*. Previously known single-nucleotide polymorphisms (SNPs) were identified by comparison with the GeneCards database, which includes the dbSNP, and removed from further analysis (http://www.genecards.org; Weizmann Institute of Science, Rehovot, Israel). The novel variants described as mutations in *FKBP10* are not found in the dbSNP, presently the most comprehensive SNP database, indicating that they have not been found previously unless otherwise noted. We have employed the conventional coding sequence numbering in which the A of the initiator methionine codon (AUG) is the first nucleotide (+1), and the initiator methionine is the first amino acid (+1). To aid in interpretation, this is identical to the coding system employed by Alanay and colleagues.(6)

## Clinical Vignettes

**Fig. 2:**
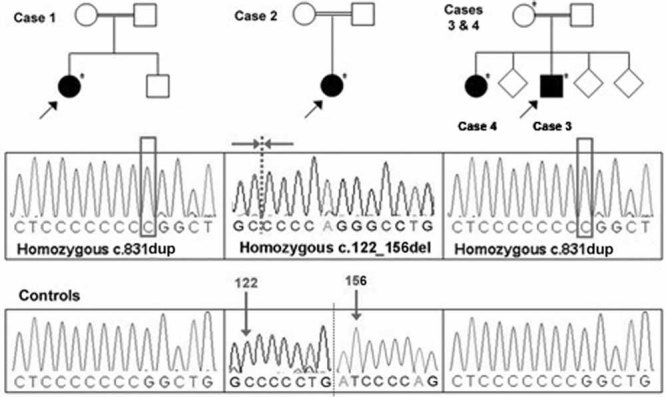
Sequencing results of *FKBP10* mutations (cases 1 through 4). Patient pedigrees are aligned above patient chromatograms, demonstrating their respective mutation in *FKBP10*. Below the patient chromatogram is a control chromatogram aligned to depict wild-type DNA sequence at the position of patient mutations. Cases 1 and 2 and siblings, cases 3 and 4, are depicted. Red boxes represent nucleotide base pair insertions. Dashed red lines represent junctional sites, where a deletion has occurred (*red arrows* indicate direction of junctioning). Asterisks (^*^) depict patients with DNA available for sequencing. The mutation for each patient is listed beneath his or her respective chromatogram.

**Fig. 3:**
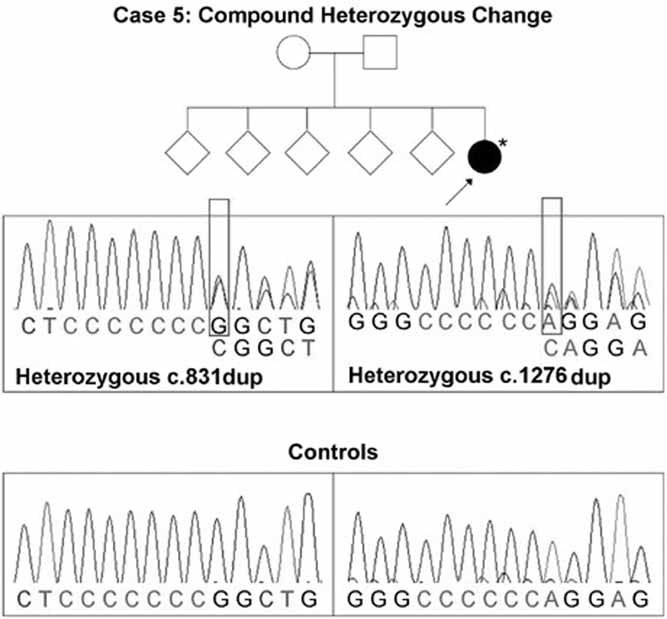
Sequencing results of *FKBP10* mutations in a compound heterozygote (case 5). The pedigree of case 5 is aligned directly above her chromatograms, demonstrating two separate mutations in *FKBP10*. The first change (*left*) is a single-nucleotide base-pair duplication (c.831dup) that results in a frameshift mutation. The second (*right*) is a single-nucleotide base pair duplication (c.1276dup) that results in a frameshift mutation. Below the patient chromatogram is a control chromatogram aligned to depict a wild-type DNA sequence at the exact position of the patient mutations.

**Fig. 4:**
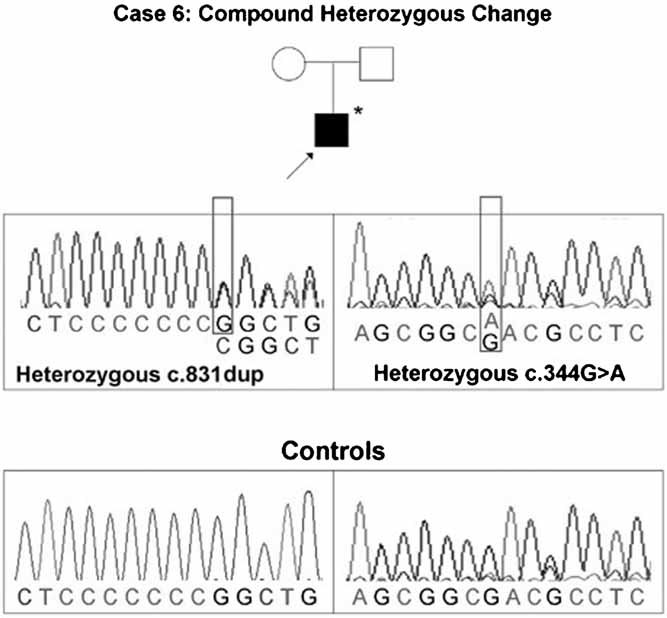
Sequencing results of *FKBP10* mutations in a compound heterozygote (case 6). The pedigree of the patient in case 6 is aligned directly above his chromatograms, demonstrating two separate mutations in *FKBP10*. The first change (*left*) is a single-nucleotide base pair duplication (c.831dup) that results in a frameshift mutation. The second (*right*) is a single-nucleotide missense mutation (c.344G > A) that results in an amino acid change p.Arg115Gln and was excluded from 100 control samples. Below the patient chromatogram is a control chromatogram aligned to depict wild-type DNA sequence at the exact position of the patient mutations.

Among the 67 total cases screened for mutations in *FKBP10*, we identified five unrelated families with six affected individuals. The clinical history of the patient in case 5 has been included in two previous publications.(20,21) Also, the clinical histories of the two patients in case 3 and case 4 have been reported previously in 2005.(20) The four patients identified in cases 1 through 4 had homozygous mutations (Fig. 2), whereas the patients in cases 5 and 6 demonstrated compound heterozygous mutations (Figs. 3 and 4).

### Case 1

**Fig. 5:**
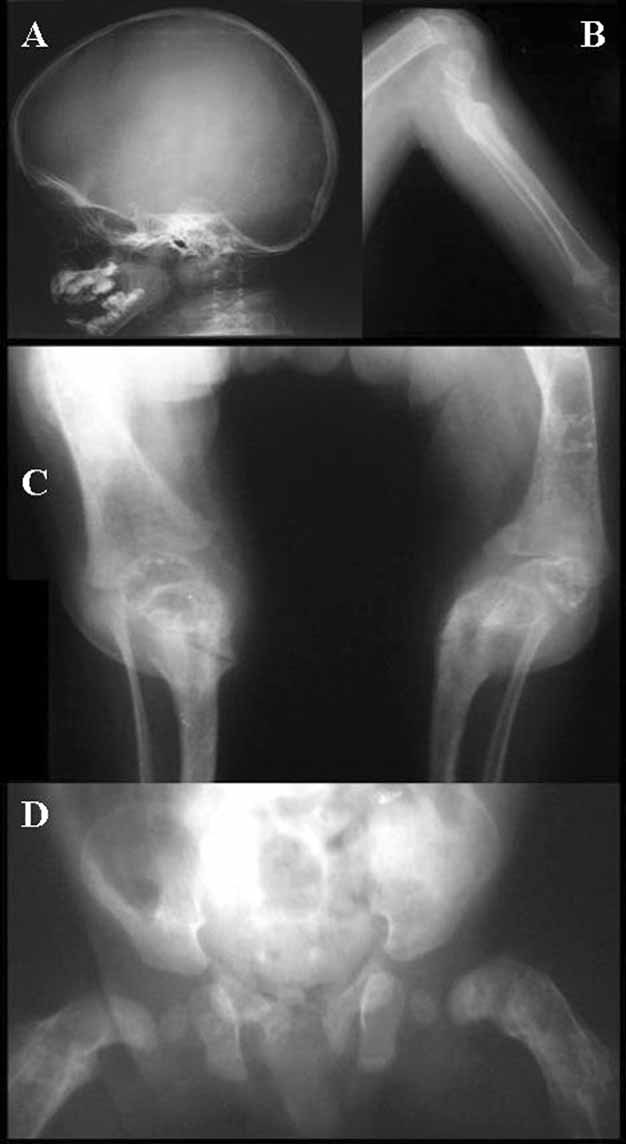
Radiographic features of an affected proband with a mutation of *FKBP10*. Plain radiographic films demonstrate the patient in case 1 at 7 years of age. Her skull (*A*) shows evidence of osteopenia and wormian bones. Her upper and lower limbs (*B*, *C*) show evidence of contractures and deformity owing to recurrent fractures. The pelvis (*D*) shows the proximal femurs with osteopenia, regional lucencies, and deformity.

This patient is the only daughter of a consanguineous Turkish marriage (first-generation cousins). She has one unaffected sibling, and both parents were asymptomatic. She presented at age 6 years with a history of bilateral congenital flexion contractures of the knees with internal deviation and bilateral extension contracture of the ankles (Fig. 5). Additionally, she had a triangular face and somewhat rhizomelic proximal extremities. Notably, her dentition, hearing, intelligence, and sclerae were normal. Beginning at 2 months of age, she experienced multiple recurrent femoral and costal fractures. At the age of 9 years, her limb movement was found to be greatly restricted, with upper better than lower extremities. Further, she was able to ambulate only by wheelchair.

In addition to her clinical history, the patient's past laboratory history included biochemical analysis of type I collagen, which demonstrated no obvious quantitative or qualitative abnormalities. Additionally, given the presence of congenital contractures, she was assayed for urine lysyl hydroxylation defects. A positive urine screen prompted screening for *PLOD2* mutations. However, no *PLOD2* mutations were discovered. Sequencing of *FKBP10* revealed a homozygous 1-base-pair duplication (c.831dup), which is the same as reported by Alanay and colleagues. This mutation is predicted to lead to a frameshift mutation and premature protein truncation (Fig. 2).

### Case 2

This patient is the daughter of consanguineous unaffected Indian Punjabi parents. She was born at term, with a length of 50 cm, weight of 3200 g, and an occipitofrontal circumference (OFC) of 34 cm. Her developmental milestones were normal. There was no notation of congenital contractures. At the age of 3 years, she sustained five fractures following minor trauma, including two fractures of the humerus, as well as fractures to the mandible, tibia, and ankle. At 5 years of age, she presented to us for clinical evaluation with failure to thrive. She was noted to have bluish sclerae and suffered growth retardation (length was less than the 25th percentile at age 1 year, less than the 20th percentile at age 3 years, less than the 1.5th percentile at age 6 years). At the time of the most recent clinical assessment, she was 22 years of age. X-rays reveal multiple wormian bones, kyphoscoliosis, and gracile fibulas. She has short stature (136 cm, –4 SD), but her sclerae are no longer bluish. She has no notable contractures. Sequence analysis for *FKBP10* revealed a homozygous 35-base-pair deletion (Fig. 2) resulting in a frameshift and premature stop codon p.(Leu41Glnfs*22).

### Case 3

This patient is the third child of consanguineous South African Venda parents (second-generation cousins). Neither parent was affected, nor were three siblings. The patient had one sister, gML, who is described as case 4. At birth, the patient was found to be growth restricted, with a length of 39 cm, weight of 2580 g, and an OFC of 35.5 cm (less than 3rd percentile). He was given an initial diagnosis of arthrogryposis multiplex congenita owing to congenital contractures of the bilateral elbows, wrists, and knees and a bilateral thumb-in-palm deformity. He had pterygia of the knees bilaterally. Beginning at 2 months of age, he suffered multiple recurrent fractures of the extremity long bones following trivial trauma, and radiographs eventually demonstrated wormian bones. He had normal sclerae and dentition. Based on the findings of congenital contractures and brittle bone disease, a diagnosis of Bruck syndrome was made. Sequencing of *FKBP10* revealed the same homozygous 1-base-pair duplication (c.831dup) as seen in case 1 and described by Alanay and colleagues (Fig. 2).

### Case 4

This patient is the elder sister of the patient in case 3. No congenital contractures have been noted in her history. Beginning at 2 months of age, she suffered multiple recurrent fractures of the long bones of the extremities, including bilateral femurs, left humerus, and left tibia. She was of notably short stature, with normal sclerae, normal dentition, and a triangular facies. At the age of 4 years, wormian bones were discovered on skull radiographs. She was diagnosed at that time with OI type 3. At the age of 10 years, she presented with marked growth retardation and was 104 cm in height (less than 3rd percentile) and 20 kg in weight. She had normal hearing and intelligence. Sequencing of *FKBP10* revealed a homozygous 1-base-pair duplication (c.831dup), as in her sibling (Fig. 2).

### Case 5

This patient is the only affected daughter of nonconsanguineous South African parents, with five normal siblings. She was diagnosed with “atypical” arthrogryposis multiplex congenita shortly after birth owing to the presence of congenital contractures. The diagnosis was later changed to Bruck syndrome because of recurrent fractures typical of OI type 3. She suffered multiple fractures of the long bones of both the upper and lower extremities. Further, radiographs demonstrated the presence of wormian bones in the skull. After reaching skeletal maturity, her fracture rate reportedly decreased. However, she suffered multiple fractures of the right tibia to the age of 27 years. At last follow-up, she was 31 years of age with limited mobility. She was employed full-time in a clerical position with relative independence using crutches and a wheelchair. Ambulation remained very difficult, and gait with crutches remained abnormal. Sequence analysis for *FKBP10* mutations demonstrated a compound heterozygous genotype. In addition to an insertion in exon 5 p.(Gly278Argfs*95), a mutation locus that was described previously by Alanay and colleagues in a different patient, she had a second single-nucleotide duplication (c.1276dup) resulting in a frameshift mutation p.(Glu426Argfs*54) and premature protein truncation in exon 8 (Fig. 3).

### Case 6

This patient is the first child of a healthy nonconsanguineous couple of Caucasian origin. She was born following a normal pregnancy and found to have bilateral clubfeet and multiple congenital contractures at birth. During the first weeks of life, she suffered multiple pathologic fractures. Clinical examination at 16 months of age revealed a relatively short stature (76 cm, between the 3rd and 10th percentile), a weight of 9950 g (10th percentile), and head circumference of 46.5 cm (between the 3rd and 10th percentile). At 5 years of age, she presented for clinical evaluation of failure to thrive. Despite a normal skeletal survey, apart from multiple wormian bones on skull X-rays, because of her personal history of several bone fractures, a provisional diagnosis of OI was given. She had brachycephaly and a triangular face. Her sclerae were white, and there were no signs of dentinogenesis imperfecta. She demonstrated mild contractures of the knees bilaterally and pedes plano valgi. Biochemical analysis of type I collagen and molecular analysis of the *COL1A1* and *COL1A2* genes were normal. Evaluation of the *FKBP10* gene revealed compound heterozygous mutations: a nonsense p.(Gly278Argfs*95) and a missense p.(Arg115Gln) mutation (Fig. 4).

## Discussion

In contrast to the patients with *FKBP10* mutations described by Alanay and colleagues, the patients presented in these studies demonstrated a high incidence of congenital large joint contractures consistent with the diagnosis of Bruck syndrome. Bruck syndrome is an OI-like syndrome consisting of osteopenia, recurrent fractures, and congenital joint contractures. Bruck syndrome is named after the German physician of the same name who reported a patient with OI who secondarily developed large joint contractures later in life. It has been suggested previously that the name *Bruck syndrome* is in fact erroneous because of the absence of congenital contractures in Dr Bruck's original case.(24) Bruck syndrome has been linked independently to chromosome 17, possibly region 17p12, and 3q23-24, representing type 1 and type 2 Bruck syndrome, respectively. Type 2 Bruck syndrome has been associated with mutations in the *PLOD2* gene, whereas the etiology of type 1 Bruck syndrome has been unknown. *FKBP10* is localized to chromosome 17q21.2 different than the preliminary localization for the original type I Bruck syndrome family. However, because only chromosomes other than 17 were definitively excluded in that family, whether FBKP10 accounts for type I Bruck syndrome there will depend on direct testing of that family. Here we present five independent probands diagnosed with Bruck syndrome. These patients were found to be negative for mutations in the *PLOD2* gene. In each case, we identified mutations in the coding region of *FKBP10*, demonstrating that mutations in this gene may be responsible for a moderately severe recessive OI phenotype with congenital contractures. Interestingly, the siblings reported in cases 3 and 4 were diagnosed with different diseases based on current classification of brittle bone disease. Further, case 2 also was diagnosed with OI. These case phenotypes underscore the difficulty in relating clinical diagnoses to molecular defects. Such variability has not been noted so far for *PLOD2*-associated Bruck syndrome type 2, where several affected individuals have had a consistent phenotype of congenital pterygia associated with moderate to severe bone fragility.(24)

Patients with *FKBP10* mutations present a phenotype characterized by a variable degree of bone fragility with or without (congenital) contractures. This fact indeed suggests that Bruck syndrome falls within the spectrum of OI phenotypes and is not a distinct biochemical disorder, which is illustrated in the patients in the cases presented here, who show both inter- and intrafamilial variability in presentation. The OI phenotype in all patients with *FKBP10* mutations reported so far is milder than the severe-to-lethal recessive OI phenotypes caused by mutations in the genes of the prolyl-3-hydroxylation complex (*CRTAP, LEPRE1,* or *PPIB*). Moreover, the phenotypes of the patients who have reached adulthood seem to improve and appear also generally milder than OI type 3 caused by classic autosomal dominant *COL1A1/COL1A2* mutations.

The addition of *FKBP10* and *SERPINH1* to the spectrum of OI-causing genes further adds to our knowledge of collagen posttranslational processing and emphasizes the complexity of dynamic bone formation and function. Again, further research is needed to clarify the full spectrum of OI and the role of collagen modifiers and chaperones in OI development.

## References

[1] Morello R, Bertin TK, Chen Y (2006). *CRTAP* is required for prolyl 3- hydroxylation and mutations cause recessive osteogenesis imperfecta. Cell..

[2] Marini JC, Cabral WA, Barnes AM (2010). Null mutations in *LEPRE1* and *CRTAP* cause severe recessive osteogenesis imperfecta. Cell Tissue Res..

[3] van Dijk FS, Nesbitt IM, Zwikstra EH (2009). *PPIB* mutations cause severe osteogenesis imperfecta. Am J Hum Genet..

[4] Baldridge D, Schwarze U, Morello R (2008). *CRTAP* and *LEPRE1* mutations in recessive osteogenesis imperfecta. Hum Mutat..

[5] Christiansen HE, Schwarze U, Pyott SM (2010). Homozygosity for a missense mutation in *SERPINH1*, which encodes the collagen chaperone protein HSP47, results in severe recessive osteogenesis imperfecta. Am J Hum Genet..

[6] Alanay Y, Avaygan H, Camacho N (2010). Mutations in the gene encoding the RER chaperone FKBP65 produce autosomal recessive osteogenesis imperfecta. Am J Hum Genet..

[7] Bank RA, Robins SP, Wijmenga C (1999). Defective collagen crosslinking in bone, but not in ligament or cartilage, in Bruck syndrome: indications for a bone-specific telopeptide lysyl hydroxylase on chromosome 17. Proc Natl Acad Sci U S A..

[8] Eyre DR, Paz MA, Gallop PM (1984). Cross-linking in collagen and elastin. Annu Rev Biochem..

[9] Ronziere MC, Berthet-Colominas C, Herbage D (1985). Low-angle X-ray diffraction analysis of the collagen-proteoglycan interactions in articular cartilage. Biochim Biophys Acta..

[10] Reiser K, McCormick RJ, Rucker RB (1992). Enzymatic and nonenzymatic cross-linking of collagen and elastin. FASEB J..

[11] Robins SP (1982). Analysis of the crosslinking components in collagen and elastin. Methods Biochem Anal..

[12] Eyre DR, Koob TJ, Van Ness KP (1984). Quantitation of hydroxypyridinium crosslinks in collagen by high-performance liquid chromatography. Anal Biochem..

[13] Breslau-Siderius EJ, Engelbert RH, Pals G, van der Sluijs JA (1998). Bruck syndrome: a rare combination of bone fragility and multiple congenital joint contractures. J Pediatr Orthop B..

[14] van der Slot AJ, Zuurmond AM, Bardoel AF (2003). Identification of PLOD2 as telopeptide lysyl hydroxylase, an important enzyme in fibrosis. J Biol Chem..

[15] Davis EC, Broekelmann TJ, Ozawa Y, Mecham RP (1998). Identification of tropoelastin as a ligand for the 65-kD FK506-binding protein, FKBP65, in the secretory pathway. J Cell Biol..

[16] Zeng B, MacDonald JR, Bann JG (1998). Chicken FK506-binding protein, FKBP65, a member of the FKBP family of peptidylprolyl cis-trans isomerases, is only partially inhibited by FK506. Biochem J..

[17] Ishikawa Y, Vranka J, Wirz J, Nagata K, Bachinger HP (2008). The rough endoplasmic reticulum-resident FK506-binding protein FKBP65 is a molecular chaperone that interacts with collagens. J Biol Chem..

[18] Ishida Y, Kubota H, Yamamoto A, Kitamura A, Bachinger HP, Nagata K (2006). Type I collagen in Hsp47-null cells is aggregated in endoplasmic reticulum and deficient in N-propeptide processing and fibrillogenesis. Mol Biol Cell..

[19] Drogemuller C, Becker D, Brunner A (2009). A missense mutation in the SERPINH1 gene in Dachshunds with osteogenesis imperfecta. PLoS Genet..

[20] Mokete L, Robertson A, Viljoen D, Beighton P (2005). Bruck syndrome: congenital joint contractures with bone fragility. J Orthop Sci..

[21] Viljoen D, Versfeld G, Beighton P (1989). Osteogenesis imperfecta with congenital joint contractures (Bruck syndrome). Clin Genet..

[22] Byers PH, Cole WG, Royce PM, Steinmann BU (2002). Osteogenesis Imperfecta. Connective tissue and it heritable disorders: molecular, genetic, and medical aspects.

[23] Marini JC, Forlino A, Cabral WA (2006). Consortium for osteogenesis imperfecta mutations in the helical domain of type I collagen: regions rich in lethal mutations align with collagen binding sites for integrins and proteoglycans. Hum Mut..

[24] Ha-Vinh R, Alanay Y (2004). Phenotypic and molecular characterization of Bruck syndrome (osteogenesis imperfecta with contractures of the large joints) caused by a recessive mutation in PLOD2. Am J Med Genet A..

